# Gastro-Enterostomy

**Published:** 1890-07

**Authors:** Seth M. Morris

**Affiliations:** Student


For Daniel's Texas Medical Journal.

### GASTRO-ENTEROSTOMY FOR CANCER OF THE STOMACH, SUCCESSFULLY DONE.

OPERATION BY DR. W. T. BULL, AT NEW YORK HOSPITAL, N. Y.

THE patient was a married woman, age 27. Four months previous to this time, she began to have attacks of nausea and vomiting, with alternating constipation and diarrhœa. She became anæmic, and lost flesh and strength. Examination revealed the presence of a large irregular quadrilateral tumor in the region of umbilicus. It was freely movable and presented characteristics of a movable kidney, which it was supposed to be- For purpose of diagnosis, an abdominal section was made when it was found to be a carcinoma of the stomach.

The abdominal wall was closed by cat gut sutures through the peritoneum, and silk through the muscles, fascia and skin.

About five weeks afterwards an operation was undertaken for her relief. The tumor was exposed by a vertical incision. It was found to occupy the pyloric half of the greater curvature of the stomach, and extended up on its anterior and posterior surfaces nearly as far as the lesser curvature. Its situation and size made it impossible to remove the growth and sew up the opening left, so the only thing to be done was to excise that half of the stomach; that was soon done.

Then to unite the duodenum to the rest of the stomach would have been very difficult, and would have likely taken too much time as the patient was in poor condition; so it was decided to effect an anaseomosis between the small intestine and the stomach.

The opening in the later was then closed by turning in its edges so as to bring the peritoneal surfaces in apposition and a number of Lembert sutures put in. The opening in the duodenum was treated in the same manner. A two inch incision was then made in the greater curvature of the stomach and a similar one in an adjacent loop of the jejunum. By means of the cat gut rings of Dr. Abbe of this city, these openings were brought opposite one another with the peritoneal surfaces in contact, when Lembert sutures were put in, thus stitching the gut to the stomach.

The abdominal wound was closed as in the previous operation. The anastomosis was effected in twenty-five minutes. At one time the patient presented symptoms of collapse; but as the operation proceeded she rallied. Five hours after the operation she vomited, bringing up a little mucus. It was followed by no ill effects, indicating that the sutures had not given way. She was fed per rectum for five days, and given fluid food by the stomach during the next nine days. At the expiration of the two weeks she was considered out of danger and was allowed solid food as it was believed that the adhesions were strong enough to withstand vomiting in case it should be caused by solid diet. The patient has now, some weeks afterwards, apparently fully recovered. The operation lasted three and one-half hours.

The urine which was perfectly normal before the operation was found after it, to contain a large number of granular and hyalinle casts, showing that a decided congestion of the kidneys was brought about by the prolonged etherization.

A gastro-enterostomy similar to this was done by Dr. Weir early in the winter with equally good results. There are not enough of these cases on recond, yet to say whether the opening made in this way will remain patent or not.

SETH M. MORRIS.

### Society Notes.

THE Louisianian State Medical Association has been compelled to postpone indefinitely their meeting, which was to have been held in June. This is in consequence of the damage done by the recent overflow, by which crops were destroyed, and consequently there is no money in the country. We tender our sympathies to our sister Society; it is a misfortune and we regret to chronicle the fact.

THE Northwest Texas Medical Society was organized at Abilene, Texas, June 7, and the following officers were elected:

President, S. H. Stout, M. D., A. M., LL. D., Cisco.
1st Vice-President, C. F. Paine, M. D., Comanche.
2nd " —. Hollis, M. D., Anson.
3rd " Jas. Isbell, M. D., Abilene.
Sec'y and Treas., B. C. Coleman, M. D., Colorado.

The first quarterly meeting will be held at Cisco, the first Tuesday in October. It is expected that the membership will reach fifty at the next meeting, as there is a large amount of good material available, and out of reach of other Associations. The Association is auxilliary to the State Association, and adopts the National Code of Ethics.

Thus the good work goes on, and the JOURNAL rejoices in seeing the fruits of its labors thus made manifest.

TAYLOR COUNTY MEDICAL SOCIETY.—This new and active Association met at Abilene, June 7, with a full attendance. The subject of dysentery was discussed by Drs. Boyce, Rodman, Harrington, Moody, Henslee, Payne, Hollis, Keown, Wallace and

Isbell. The discussion covered the entire ground; some favoring one and some another treatment. The JOURNAL has been favored by the courteous Secretary, Dr. Hicks Florence, with a summary of the debate; but in as much as we publish herewith the same subject, as discussed before Austin District Society, and as there were no new ideas brought out, i. e., nothing different in substance from that given herewith, it is omitted. The occasion was availed of to effect an organization of the Northwest Texas Medical Association. The details of this new organization appear in this number.

---

THE SEVENTEETH ANNUAL SESSION of the Mississippi Valley Medical Association will be held at Louisville, October 8th, 9th, and 10th, 1890. The medical profession is respectfully invited to attend. The meeting promises to be of great social and scientific interest, as the profession of Louisville are doing their utmost to make it a success. Ladies accompanying physicians will be made especially welcome. Gentlemen wishing to read papers will please send titles of the same at an early date to the Secretary, Dr. E. S. McKee, Cincinnati.

---

THE WEST TEXAS MEDICAL ASSOCIATION will hold its fifth quarterly session at San Antonio, July 30, inst., Dr. G. W. Kerr, President; Dr. D. Berry, Secretary. This society knows how to combine business with pleasure; and always "adjourns from labor to refreshments." The JOURNAL acknowledges the courtesy of an invitation to the banquet, and will endeavor to be represented on the occasion.

---

SAN ANTONIO, July 1st, '90.

DEAR DOCTOR:—You are cordially invited to attend the Fifth Quarterly Session of the West Texas Medical Association, to be held at San Antonio, Wednesddy July 30th, '90, at Sholtz Hall. The meeting will be held at 10 A. M., 3:30 P. M. and 8:30 P.M. Papers will be presented by the following gentlemen: Dr. N. A. Tutwiler, of Flatonia, on Hæmorrhagic Malarial Fever; Dr. W. Boll, of Castroville, on Septicæmia; Dr. B. F. Kingsley, on Subinvolution; Dr. P. W. Johns, on Diagnosis of Pregnancy; Dr.

N. J. Trollinger, on Hay Fever; Report of a Case of Laparotomy for removal of a Fibro Cystoma, by Dr. D. Berry. One meeting of the session will be devoted to the discussion of Medico-Legal topics. Please let me know at once if you can attend.

D. BERRY, Sec y.

G. W. KERR, M. D., Pres.

---

THE Central Texas Medical Association held a very profitable session at Waco on the 8th and 9th, inst.

---

Drainage Tubes in the Bladder for Retention of Urine. —The Medical Review, of May 31, contains an abstract of an article written by Dr. Lowe, (Br. Med. Association), in which two cases of retention of urine—one from prostatic enlargement, the other from atony of the bladder—were treated, after fruitless attempts to pass the catheter, by puncturing the bladder over the pubis with a small trocar and passing rubber tubing through the canula into the bladder. Four inches were thus passed, the other end of the tube was conducted into a receptacle underneath the bed. A syphon action was established and the bladder was thoroughly drained. In one of the cases the tube was left in the bladder ten days, when it was ascertained that a catheter could be passed with ease and the tubing was withdrawn. In the other case the tube was left in the bladder for several weeks. The following were the advantages claimed: 1. That in spite of the small size of the tubing the bladder was thoroughly drained. 2. There was no escape of urine by the side of the tube. 3. The aperture caused by the tube was so small as to heal up without any difficulty or escape of urine through it. The success of the treatment in at once relieving the severe constitutional symptoms in the case of atony was very marked and complete.

---

### The Sample Copy Fiend.

spring Lake, ju 2

Mr editur sir pleas Send Me A Samp Copey of You danial med joul with a View of subscribing i am taking 4 & o Bligfrat
B Horst m. D

